# Development of FT-NIR Models for the Simultaneous Estimation of Chlorophyll and Nitrogen Content in Fresh Apple (*Malus Domestica*) Leaves

**DOI:** 10.3390/s150202662

**Published:** 2015-01-26

**Authors:** Elena Tamburini, Giuseppe Ferrari, Maria Gabriella Marchetti, Paola Pedrini, Sergio Ferro

**Affiliations:** 1 Department of Life Sciences and Biotechnology, University of Ferrara, Via L. Borsari 46, Ferrara 44121, Italy; E-Mails: mhm@unife.it (M.G.M.); pdp@unife.it (P.P.); 2 BÜCHI Italia S.r.l., Via Galileo Galilei, 34, Cornaredo (MI) 20010, Italy; E-Mail: ferrari.g@buchi.com; 3 Department of Chemical and Pharmaceutical Sciences, University of Ferrara, Via Fossato di Mortara, 17-27, Ferrara 44121, Italy; E-Mail: fre@unife.it

**Keywords:** FT-NIR, Total Kjeldhal Nitrogen, chlorophyll, fresh leaves, apple, fertilization

## Abstract

Agricultural practices determine the level of food production and, to great extent, the state of the global environment. During the last decades, the indiscriminate recourse to fertilizers as well as the nitrogen losses from land application have been recognized as serious issues of modern agriculture, globally contributing to nitrate pollution. The development of a reliable Near-Infra-Red Spectroscopy (NIRS)-based method, for the simultaneous monitoring of nitrogen and chlorophyll in fresh apple (*Malus domestica*) leaves, was investigated on a set of 133 samples, with the aim of estimating the nutritional and physiological status of trees, in real time, cheaply and non-destructively. By means of a FT (Fourier Transform)-NIR instrument, Partial Least Squares (PLS) regression models were developed, spanning a concentration range of 0.577%–0.817% for the total Kjeldahl nitrogen (TKN) content (R^2^ = 0.983; SEC = 0.012; SEP = 0.028), and of 1.534–2.372 mg/g for the total chlorophyll content (R^2^ = 0.941; SEC = 0.132; SEP = 0.162). Chlorophyll-a and chlorophyll-b contents were also evaluated (R^2^ = 0.913; SEC = 0.076; SEP = 0.101 and R^2^ = 0.899; SEC = 0.059; SEP = 0.101, respectively). All calibration models were validated by means of 47 independent samples. The NIR approach allows a rapid evaluation of the nitrogen and chlorophyll contents, and may represent a useful tool for determining nutritional and physiological status of plants, in order to allow a correction of nutrition programs during the season.

## 1. Introduction

Nitrogen (N) plays a fundamental role in crop production and is a major tool for controlling growth and fruiting in agriculture [1,2]. Unfortunately, owing to the intensive use of N-based fertilizers, the levels of nitrates in groundwater are often above the safe thresholds proposed by US-EPA [3] or EU [4], thus representing a threat to human health [5]. Because of their detrimental effects on the environment, methods of treatment and prevention have to be considered in order to protect soil and groundwater from nitrate leaching, thus preventing the latter from reaching high concentrations [6,7], but at the same time assuring high yields and optimal quality for the crop [8]. In fact, a too low supply of N to the plants may induce a physiological stress and a substantial reduction of the photosynthetic activity of leaves [9,10], with dramatic effects on crop production.

On the other hand, it is well known that chlorophyll is an essential pigment for the conversion of light into chemical energy. From a chemical point of view, it is a large porphyrin molecule with a magnesium atom at its center and a long hydrocarbon chains that serves to anchor the molecule to the inner membranes of the chloroplast. Actually, chlorophyll in leaves is a mixture of two compounds: chlorophyll-a (Chl-a) and chlorophyll-b (Chl-b). The former is the pigment that participates directly to the light-based photosynthetic reactions, while the latter is an accessory pigment that takes part to the photosynthesis indirectly, by transferring the absorbed light to Chl-a. The two molecules differ only in one of the functional groups bonded to the porphyrinic ring (in Chl-b, there is an aldehydic group in place of a methyl group) [11].

From an applied perspective, a quantification of Chl content in leaves is important for both land managers and agronomists, because pigmentation can be directly related to physiology and the state of stress of plants [12]. Foliar N-content determination is useful as well, because a large portion of N is located within enzymes in chloroplast and mitochondria that are responsible of several leaf functions, thus affecting the growth of the tree, as well as the yield and quality of fruits [13].

Conventional wet chemical methods for the determination of N and Chl foliar contents typically require laborious and destructive approaches, such as the so-called Kjeldahl method for total N determination [14], and the extraction with solvents (e.g., DMSO, acetone) followed by spectrophotometric determination, in the case of chlorophyll [15]. Moreover, the above analysis are both expensive and time-consuming, and do not allow repeated measurements on individual leaves, directly in the field [16].

Recently, exploiting the strong color characteristics of Chl, non-destructive hand-held optical instruments have been developed [17]. They are based on measuring the difference in light transmission at red (650 nm) and infrared (940 nm) wavelengths. Unfortunately, they often provide only a relative and not reproducible estimation of the Chl concentration [18,19]. Owing to the fact that more than one-half of the leaf N is located in the photosynthetic apparatus, correlations have been attempted between optical readings and nitrogen content, but results were not satisfactory [20].

In recent years, remote sensing techniques and fluorescence imaging have also been developed, which are based on the amount of radiation reflected by the leaf coverage [21,22]. Whilst promising results have been obtained, the latter approaches seem to be suited only to large areas, typical of extensive agriculture (*i.e.*, cereal crops).

Therefore, a reliable and interactive approach to nutrition of fruit trees, allowing to monitor the plants' nutrient request and the physiological status of trees during the growing season, thus permitting a proper fertilization program, based on results obtained, is currently desirable but not available yet.

NIRS-based technology is widespread in different industrial sectors, from pharmaceuticals [23] to food [24] and bioprocesses [25]. Up to now, applications of NIR to orchards have been only focused on the analysis of apple-fruit firmness, ripening and sugar content, or they have been oriented to post-harvest quality control, in combination with visible spectroscopy but only exploiting the narrow range 12,500–10,000 cm^−1^ [26,27]. In addition, several NIR applications have dealt with N monitoring in fertilized crops [28–30], but none of them has been associated with the simultaneous determination of Chl.

Since NIR permits the non-destructive and real-time measurement of several parameters at a time, assuring reliable results and robust predictive ability, it may provide a way to overcome the drawbacks related to the use of the traditional analytical approaches.

In recent years, the NIR approach has been widely recognized as one of most promising on/in line detection methods in agri-food and other areas [31]. Industries involved with foods have traditionally used NIR measurements for quality control, blending, and process control [32]. Developments in computer science and chemometrics have allowed parallel improvements in the on/in-line NIR techniques, and have attracted considerable attention from researchers in agriculture. For example, the approach was successfully applied for measuring chemical constituents such as protein, starch, fiber and moisture in agricultural products [33] and for detecting soil properties such as organic matter, minerals, texture, nutrients, water, pH, and heavy metals [34].

The present research aims at proposing a NIR-based approach for the quantitative evaluation of the concentration of both, N and Chl (total, and of the two forms: Chl-a and Chl-b) in fresh leaves. An apple orchard has been used as a case study, since a number of scientific experiments have confirmed that apple trees, in particular, need a tailored N fertilization program [35,36]. Accordingly, the development of a spectroscopy-based method, which allows the simultaneous and direct determination of actual contents of both Chl and N in fresh leaves, could provide the desired nutritional and diagnostic information, during the growing season. Within this paper, the results of preliminary investigations on the possibility to determine nitrogen and chlorophyll in fresh leaves will be presented; such a research represents the starting point for subsequent studies on the capability of NIRS-based portable instruments to direct and real-time monitor the response of plants to nitrogen fertilization.

## 2. Experimental Section

### 2.1. Apple Leaves Sampling

An orchard of *Malus domestica* cv. Imperatore Dallago, of about 30 years of age and located in the Province of Ferrara (Emilia Romagna, Italy), was chosen as experimental site. In particular, the attention was focused on a set of 30 apple trees, whose foliage has been divided into low, middle and top sections. For each section, two random samplings of five leaves were carried out. NIR spectra were collected on fresh samples immediately after picking (less than 5 min elapsed between excision of the leaf and completion of scans), thus avoiding foliar tissue degradation and changes in chemical composition. Then, samples were rapidly cooled to −80 °C in order to ensure a correct preservation of pigments and to allow subsequent off-line analyses. Samplings were carried out during summer/early fall of 2013, by collecting 180 samples. To construct the calibration models, 133 samples were used, while the remaining 47 were preserved to perform subsequent external validations.

### 2.2. NIR Modeling

NIR spectroscopy is a secondary or correlative technique, *i.e.*, the spectral data collected are correlated through statistical means to some reference (laboratory) data. It is a type of vibrational spectroscopy in the wavelengths range of 4000–10,000 cm^−1^. The spectral occurrence in the NIR region are dominated by overtones and combination bands, characterized by weak and overlapped absorptions. Although such spectra are more difficult to interpret according to conventional techniques, a remarkable amount of information can be extracted by employing chemometric techniques [37]. They have been used in order to build regression models, which are subsequently developed as prediction tools [38].

Extraction of information from NIR spectra requires a multivariate approach. The latter is a complex and very important part of NIR spectroscopy, especially for quantitative analysis. The purpose of multivariate-analysis methods is to construct models capable of accurately predicting the characteristics and properties of unknown samples. The process involves the steps described in Table 1.

Because of the vast amount of spectral information provided by NIR spectrophotometers, the substantial number of samples required to construct classification and calibration models, and the high correlation in spectra, there is a need for methods reducing the number of variables. As a result, a number of multivariate-analysis methods relies upon variable-reduction techniques that allow reducing the dimension of original data to a restricted number of uncorrelated variables, which anyway contain the relevant information. To this end, the principal component analysis (PCA) was selected [39]. PCA looks for the directions of maximum variability within sample groupings and uses them as new axes called “principal components”. In this way, the relevant information for the system is contained in a reduced number of variables. The PCA data thus obtained can be used as new variables in subsequent calculations, instead of the original data. In NIR spectroscopy, the most frequently used multivariate-regression methods are the partial least-squares (PLS) regressions. PLS can be used either in specific spectral regions or on the whole spectrum; we chose this latter option, as it allows including more information in the calibration model. PLS uses the principal components provided by PCA to perform a regression on the sample property to be predicted, finding the directions of greatest variability by considering both spectral and target-property information, with the new axes called *factors*. The optimal number of factors to be used was determined through the predicted residual error sum of squares (PRESS) calculation, which shows the sum of squares of deviations between predicted and reference values. As a rule, the number of factors depends on the complexity of the system but it should be in the range 4–13. Validation is the final step in the development of a quantitative model. Validation can be internal, extracting samples from calibration sets, or external, collecting new independent samples. External validation is always recommended because it produces more realistic results. The final model, employed for routine analysis permits to predict the property of interest in unknown samples [40].

### 2.3. NIR Spectra Collection, Treatment and Calibration

FT-NIR spectra were collected with a NIRFLex N-500 (Büchi, Flawil, Switzerland) instrument equipped with a Petri-dish drawer (Büchi). A polarization interferometer with TeO_2_ wedges was set up. Single fresh leaves were flattened on the glass surface of a Petri dish (9.0 cm of diameter), by using a stainless steel load disk. Diffuse reflectance was measured with the adaxial surface of the leaf facing the incident beam. The instrument was designed to be used under usual environmental conditions (from 5 to 35 °C), without any drift in the spectrum signal.

The reflectance spectra were recorded by using the NIRWare 1.0 software (Büchi) and scanning the whole range, from 10,000 to 4000 cm^−1^, at intervals of 8 cm^−1^. Measurements were carried out at 2–4 scans per second and with a wavenumber accuracy of ±0.2 cm^−1^ (as measured with a HF gas cell at room temperature, 25 ± 5 °C). In order to obtain a good signal-to-noise ratio, 128 scans were averaged during each spectral acquisition, resulting in a total measurement time of 30 s. To optimize the spectrum baseline, every acquisition was preceded by the registration of an internal reference. All chemometric analyses, including mathematical pretreatments, calibration and validation, were performed by means of the NIRCal 5.4 software (Büchi). In order to minimize the multiplicative interferences of scatter and surface roughness, which are generally found on diffuse reflectance spectra of solid samples, the raw optical data were processed with a combination of pretreatments based on second derivatives. In addition, for minimizing the baseline drifts and overcoming the scattering interferences caused by the non-uniformity of the leaf surface and by the presence of cuticular wax, a multiplicative scatter correction (MSC) method was applied before setting up the calibration model [41]. Pretreatments of spectra are widely used, in order to reduce light scattering, whereas MSC is a specific tool that can discriminate the informative absorbance of analytes from the scattering signal in spectral data. It can be properly used to minimize the spectral differences, within the same batch of samples, caused by the non-uniformity of materials submitted to light signal. The NIRCal software, based on correlation coefficients calculated for each wavelength, suggests the optimal wavelengths, or wavelengths intervals, for each set of data under processing. To judge of the quality of calibration results, during the iterative optimization process automatically carried out by NIRCal, the Q statistic was assessed for every intermediate calibration, considering the various combinations of wavelength ranges and data pretreatments.

The Q statistic, also known as the square prediction error, is widely used for quantifying how well a data set is represented by a model [42]. The Q value weighs up all the calibrations through a number comprised between 0 (useless) and 1 (ideal). When Q is greater than 0.75, it means that the calibration is providing reliable results. Basing on Q values, the best calibration models were obtained by using the derivative and MSC approaches in sequence (transformed spectra not shown). A value of 0.74 was obtained for the calibration of Chl-b, which is not surprising, since it is plausibly due to the statistic weakness of the model, due to the low absolute concentration values and ranges, and the high number of factors (Table 2).

To establish the relationship between NIR values and the data obtained through off-line wet-chemical analyses, a Partial Least Squares (PLS) regression was used. Outliers detection was performed by the software based on the Mahalanobis distance criterion [43]. As internal validation procedure, the so-called cross-validation, a default software output was used. To quantify the probability of autocorrelation between a given series of spectra, the Durbin-Watson (DW) test was applied to the residuals of PLS regression [44]. This statistic tool assesses the likelihood for the error values of the regression to have a first-order auto-regression component. Small values for the DW statistics indicate the presence of autocorrelation, and test results always lie between 0 and 4. If the DW outcome is substantially lower than 1.5, there is evidence of a positive serial correlation. If DW > 2.5, successive error terms are, on average, much different in value one from another, *i.e.*, they are negatively correlated, which implies an underestimation of the level of statistical significance [45]. The selection of the best quantitative regression models was carried out using squared Pearson correlation coefficient for calibration (R^2^_cal_) and cross validation (R^2^_cross val._), standard error of calibration (SEC) and standard error of cross-validation (SECV). Relative Prediction Deviation (RPD), *i.e.*, the relationship between the SD (Standard Deviation) of the entire population divided by the SEC, was also calculated for both calibrations and cross validations [46]. Williams [47] proposed the use of R^2^ together with RPD as the most meaningful statistics for appraisal of analytical efficiency by NIRS. The RPD is a simple statistics that enables the evaluation of the prediction accuracy in terms of standard deviation of reference data, providing a standardization for the SEP. It is calculated by dividing the SD of reference values in the validation by the SEP. A good model should be characterized by a SEP value much lower than the SD value, so that high RPD values are usually desired. Minimum requirements for RPD values of 2.0–3.0 for adequate screening and 3.0–5.0 for an acceptable predictability have been suggested. Values exceeding 5.0 indicate that the prediction model is almost perfect.

### 2.4. External Validations

The calibration models were validated by means of external validations: 47 of the 180 independent samples collected during the campaign were acquired to obtain additional data and evaluate the predictive capability of the calibration models. The prediction accuracy was considered in terms of squared Pearson correlation coefficient (R^2^_pred_), standard error of prediction corrected for bias (SEP_-b_), and root mean standard error of prediction (RMSEP). Residual standard deviation (RSD) and repeatability were calculated in order to evaluate NIR precision. Finally, the standard error of the laboratory (SEL), *i.e.*, the error of the reference data, was reported in order to allow a comparison between that value and the NIR performance (SEC and SEP_-b_).

### 2.5. Off-Line Reference Assays

The mass of fresh leaves was determined prior to pigment analysis. Three leaves from each sample were weighed together; then, chlorophyll pigments were extracted in ultra-pure acetone (Carlo Erba, Milano, Italy) by grinding the leaves in a mortar, and the extract absorption was measured by means of an UV/Vis Spectrophotometer (Shimadzu Europe, Duisburg, Germany) using a few fixed wavelengths (470, 645 and 662 nm). The concentrations (mg/g of fresh leaf mass) of Chl-a, Chl-b and total chlorophyll were assessed by using the equations proposed by Wellburn and Lichtenthaler [48].

For the determination of the total N content, as the sum of organic N, ammonia (NH_3_) and ammonium (NH_4_^+^), a Kjeldhal apparatus was used: grinded leaf samples (2.5 g) were boiled in 98% sulfuric acid (Merck Millipore, Darmstadt, Germany) up to complete digestion. The process was catalyzed by adding cupric sulfate tablets (Merck Millipore) and the obtained solution was then distilled in a solution of boric acid (AnalaR NORMAPUR^®^, VWR Chemicals, Radnor, PA, USA). The total amount of N originally present in the sample, was determined as ammonia by a back-titration with a 0.2 N sodium hydroxide solution (Avs Titrinorm^®^, VWR Chemicals). Results of Total Kjeldhal Nitrogen (TKN) content were expressed as percentage weighted on fresh leaf mass.

## 3. Results and Discussion

### 3.1. Fresh Leaves Spectra Characteristics

**Figure 1.**
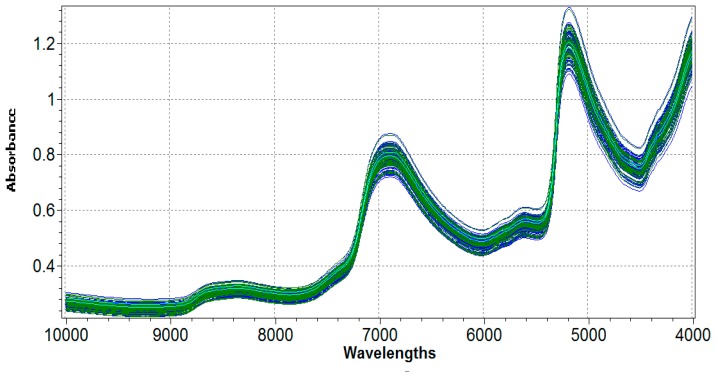
NIR raw absorbance spectra of fresh apple leaves. NIR-Cal^®^ software automatically subdivides original spectra in calibration (blue) and validation (green) sets.

Examples of original spectra are shown in Figure 1. Since spectra were collected on fresh leaves, they are dominated by a large absorption of water at around 6876 and 5150 cm^−1^, owing to the first O-H overtone and O-H combination band, respectively [49].

Typically, spectra of fresh materials do not allow an easy visual interpretation, because of the presence of several interferences. In highly complex structures, like the vegetal tissues, the combination of electrostatic and electromagnetic forces caused by the dynamic spatial arrangements of some atoms (such as carbon, oxygen, nitrogen and hydrogen) and inorganic elements (*i.e.*, magnesium in the chlorophyll molecule) can lead to significant variations in the relationships among the constituents, and consequently on spectral information. Otherwise, the general optical properties of apple fresh leaves have shown to be highly correlated with their photosynthetic performance. In the region comprised between 10,000 and 7700 cm^−1^, the spectra of the leaves do not show any particular absorption feature, and the reflectance intensity is mainly affected by structural features [50]. Typically, the presence of waxes and cuticles on the surface of the leaf generates light scattering or shift of the baseline, which may interfere with NIR spectra acquisition and requires to be corrected during the processing phase of spectra. Pigments are known to show intense absorbance in the visible region (16,000–13,600 cm^−1^). However, in the range of wavelengths closer to red (6000–4000 cm^−1^), the absorption is also characterized by some special features, which can be correlated to chlorophyll content [51,52]. Although highly overlapped and broadened, distinctive bands of chlorophyll can be recognized in the regions 4300–4800 cm^−1^ (due to methyl, methylene and -CHO combination bands) and 5600–6000 cm^−1^ (related to the -CH_2_/-CH_3_ first and second overtones), respectively.

In addition to the content located within the photosynthetic apparatus, foliar-N is mainly present within proteins. In NIR spectra, N-related signals, due respectively to the first and second overtones of amide groups (-CONHR) at 5570–4530 cm^−1^ and 6600–7100 cm^−1^, are usually difficult to identify because they are almost completely covered by water absorption [53]. Fortunately, the -CONHR combination bands (at 4300–4100 cm^−1^) as well as a weak signal related to the second or third -CONHR overtone (in the region 8500–8300 cm^−1^) can be identified on spectra set.

A full-spectrum approach was used to build the PLS calibration models (1241/1501 wavelength points). All spectral regions were used to assess correlations between the absorbance and the associated analytical information, with the only exception of a small portion between 7404 and 7144 cm^−1^, automatically excluded from calculation by the software, because not sufficiently correlated with analytical information.

### 3.2. TKN Calibration Model

**Figure 2.**
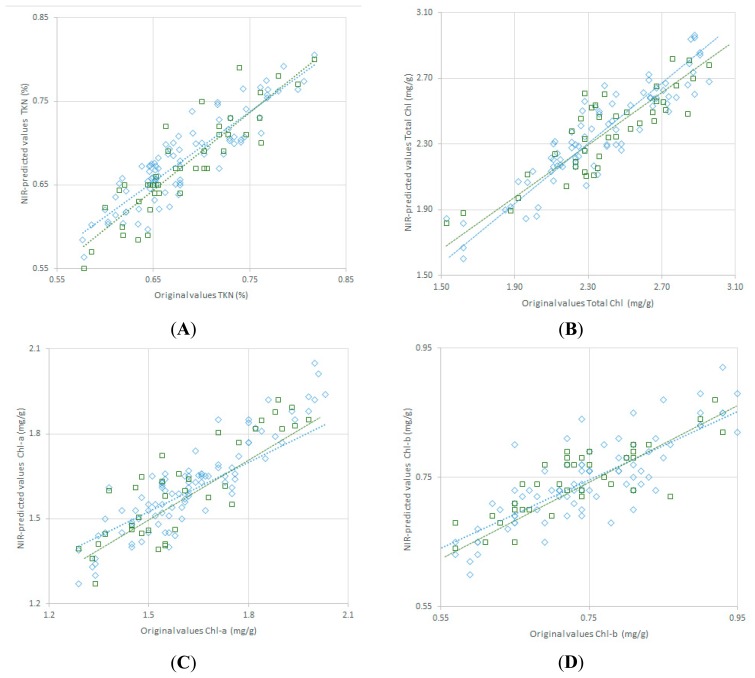
NIR-predicted *vs.* original values for TKN (**A**); total chlorophyll (**B**); Chl-a (**C**) and Chl-b (**D**). Calibration (open rhombus) and cross-validation (open squares) curves are shown. Concentrations are expressed as % of fresh leaves mass for TKN, and as mg/g of fresh leaves mass for chlorophylls.

On the 133 samples used to build the calibration models, the TKN content ranged between 0.577% and 0.817% of fresh leaves; no outliers were found in the sample set. The blockwise cross-validation was performed by the software, by randomly choosing four samples at a time from the calibration set (C-set) to be assigned to the validation set (V-set). Both the calibration curve, obtained with the PLS regression model (7 factors), and the cross-validation curve are shown in Figure 2. The standard error of calibration (SEC) and the regression coefficient (R^2^) were 0.012 and 0.983, respectively; the validation samples were predicted with a SECV of 0.016 and a R^2^ of 0.945 (see Table 2).

Based on the DW statistics, both the C-set and V-set showed no autocorrelation. RPD for calibration and cross validation were very satisfactory in terms of predictive ability of the model.

A comparison between SEL and SEC has shown an unavoidable worsening of the accuracy, when passing from reference assays to NIR results, due to the intrinsic characteristic of NIRS to be a secondary technique.

### 3.3. Chlorophyll (Total, Chl-a, Chl-b) Calibration Model

By considering 133 of the samples collected (see Table 2), separate calibration models were calculated for total Chlorophyll, Chl-a and Chl-b. Five samples were excluded in the case of the latter, while no outliers were found in the case of total Chlorophyll and Chl-a. Calibration and cross-validation regressions are shown in Figure 2. Despite the very low absolute content of chlorophyll on leaf mass (only a few milligrams per gram, especially with regard to Chl-b), and taking into account the physiologically narrow range of concentrations (see Table 2), results appear quite promising. Generally speaking, the total chlorophyll amount in leaves of non-stressed apple plants is usually in the range 0.4–4.6 mg/g, depending mainly on fertilization level and on fruit ripening [53]. The number of factors was particularly high in the case of the two chlorophyll components, Chl-a (9 factors) and Chl-b (10 factors), a fact that could compromise the predictive capability of the calibration models. Actually, regression coefficients of correlation (R^2^) lower than 0.95 were obtained for both calibration and cross-validation. For practical purposes (*i.e.*, a first attempt to directly determine Chl concentrations using exclusively the NIR spectral region), they anyway represent a promising outcome, which needs to be improved. Regarding SEC values, they were respectively equal to 0.132, 0.076 and 0.059, for total chlorophyll, Chl-a and Chl-b. As expected, also RPD values confirmed that the results obtained were at the level of screening. Similarly to TKN data, the DW statistics confirmed the absence of autocorrelation for both C-set and V-set samples (see Table 2).

### 3.4. External Validation and Prediction Capability of the Models

**Figure 3.**
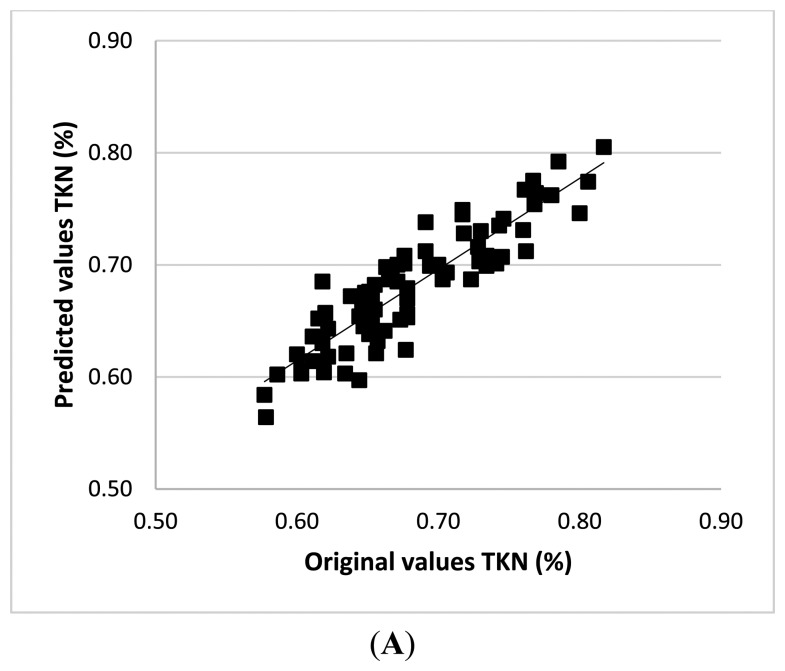

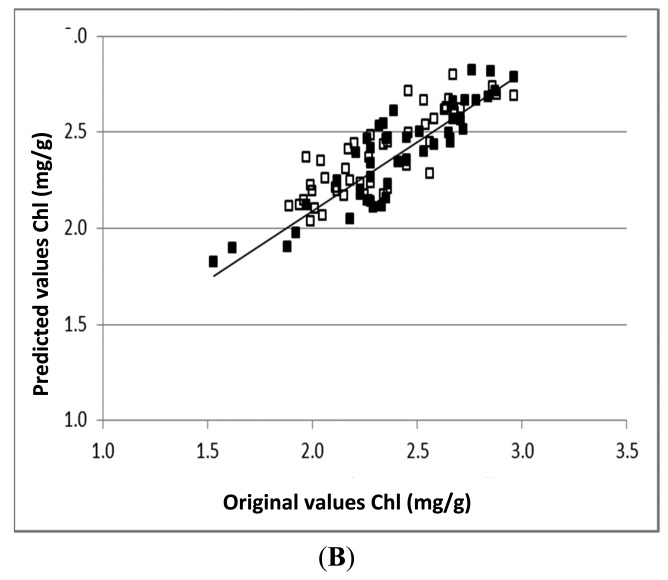
Comparison between NIR-predicted and measured values for TKN concentration (**A**); and chlorophyll content calculated as total (closed squares) and as the sum of Chl-a and Chl-b (open squares) (**B**).

External validation was carried out for all the parameters of interest, in order to evaluate the actual predictive capability and robustness of calibration models. An internal validation procedure cannot be considered a sufficient test, especially in the case of complex natural samples, which are strongly affected by physiological and nutrition status as well as by surface tissue composition and structure. Accordingly, other 47 independent leaf samples were collected, as previously described, and submitted to NIR detection and then to conventional (wet) chemical assays. The results of these supplementary tests have been reported in terms of NIR-predicted TKN concentration (Figure 3A) and total chlorophyll (Figure 3B), against the off-line analytical assay results (Table 3). Figure 3B comprises also estimates of total chlorophyll calculated as the sum of Chl-a and Chl-b predictions. Predictive capability and accuracy were quite satisfactory for TKN (R^2^ = 0.940; SEP_−b_ = 0.028) and anyway acceptable for total Chl (R^2^ = 0.899; SEP_−b_ = 0.162). As expected, worse results were obtained for the latter, owing to the unavoidable propagation of errors due to the sum operation.

The bias represents the average difference between the predicted and measured reference value, for all samples in the prediction set, and it is also used to check whether there is a systematic difference between the average values of samples in the calibration and prediction sets. In the absence of such a difference, a bias should not be detected. In predictions related to Chl-b, a non-negligible bias was observed that could arise from an overestimation of predicted values, thus affecting the RMSEP value. Nevertheless, apart from a strict statistical meaning and the possibility of further improvements, it is worthwhile noting that the non-negligible bias does not represent a limitation to an in-field application. In fact, for evaluating the response of plants to environmental stresses, the chlorophyll fluctuation, rather than its absolute value, is usually used as the valuable index. Moreover, the present approach, which makes available the independent and simultaneous determination of both chlorophyll and N, allows a sort of reciprocal internal control between obtained values.

### 3.5. Advantages of NIR for In-Field Applications

**Figure 4.**
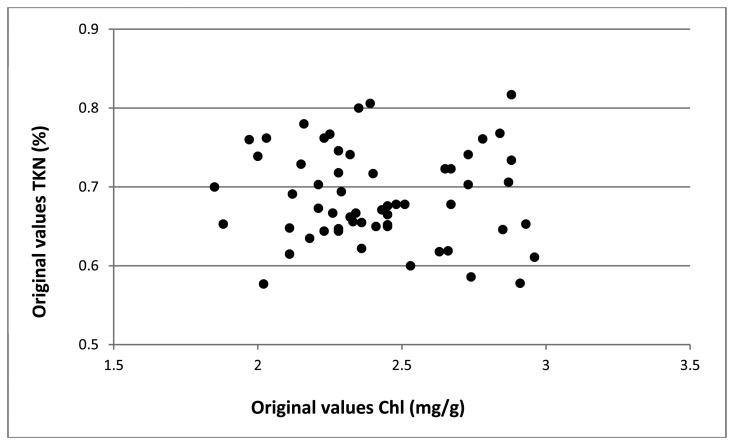
Relationship between total chlorophyll and TKN content in fresh apple leaves (data obtained through chemical analyses).

As previously mentioned, as much as 60%–75% of the TKN in a plant is located within the photosynthetic apparatus; for that reason, chlorophyll concentration can be exploited as an indirect estimation of plant N status [54]. Nevertheless, the effectiveness and reliability of this approach have been questioned [55], owing to the fact that the relationship between chlorophyll and N-content depends on growing conditions, seasonal trends, specific leaf features, nutritional status of the plant and so on. R^2^ values between 0.3 and 0.8 can be found in literature [56] for the correlation between the N-content and the foliar reflectance in the visible; moreover, the correlation dramatically falls at high values of chlorophyll. It is also worth recalling that, in our case, data on wet TKN and chlorophyll contents have shown an undeniable lack of reciprocal linear correlation (Figure 4), thus validating the weakness of the above linearity assumption.

As a main competitive advantage, the NIR method allow obtaining the direct and simultaneous evaluations of both, chlorophyll and TKN, with a good reliability as well as by-passing mathematical calculations and related propagation of errors.

In our opinion, the possibility of using a simple and rapid method for monitoring both parameters, as independent values, may represent a great step forward towards the covering of the actual lack of information. In addition, it would provide the farmers with an effective and reliable method for scheduling the times and modes of nitrogen fertilization, allowing for a daily knowledge of the physiological status of plants. Besides improving the quality and quantity of fruits, the overall fertilization management could be optimized according to the effective needs of the plant (not based on “a priori” protocols or as a consequence of farmers' past experience); savings, in terms of labor and energy costs, can also be achieved.

## 4. Conclusions and Outlook

The NIR spectroscopy has proved to represent a suitable and sensitive tool for the non-destructive and simultaneous estimation of both, chlorophyll and N-content, in fresh leaves, thus providing reliable information on the physiological status and nutritional requirements of trees. In order to perform the investigation, the NIR instrumentation has been brought very close to the field, thus minimizing the distance (spatial and temporal) from leaf sampling and spectrum acquisition. Then, calibration models have been built by using a full-spectrum approach, with the aim of obtaining as much information as possible from the spectra. As a further development, the capability of NIR to respond to different level of N fertilization has to be assessed. In fact, the proposed investigation represents only the first step to pave the way for set up an *ad hoc* handheld/portable equipment for farmers and technicians, to be used directly in the field. Once designed the prototype or starting from a commercial instrument already available, in order to achieve and maintain satisfactory calibration models, an iterative procedure will be carried out. Transferring to the portable instrument the calibration models developed in this study, new samples will be collected and used for external validation. Samples used for the new external validation will be then added to the original calibration data base, thus forming new calibration models, which will be newly validated with other separate validation sets, building each time increasingly representative and robust calibrations, until the required level of predictive ability will be reached. The use of a NIR technology can represent an impressive step forward towards the resolution of the current issues of sustainable use of resources and minimization of cost-management, even in agriculture.

## Figures and Tables

**Table 1.** Steps in the multivariate model-construction process.

**Step**		**Purpose**
1	Choosing the calibration samples.	To select a set of samples representative of the whole population.
2	Determining the target parameter by using the reference method.	To determine the value of the measured property in an accurate and precise manner. The quality of the value dictates that of the calibration model.
3	Recording the NIR spectra.	To obtain physicochemical information in a reproducible manner.
4	Subjecting spectra to appropriate treatments.	To reduce unwanted contributions (such as shifts and scatter) to the spectra.
5	Constructing the model.	To establish the spectrum–property relationship using multivariate methods.
6	Validating the model.	To ensure that the model accurately predicts the property of interest in samples not subjected to the calibration process.
7	Predicting unknown samples.	To predict rapidly the property of interest in new, unknown samples.

**Table 2.** Statistics of calibration- and cross-validation results.

**Parameter**	**TKN**	**Total Chl**	**Chl-a**	**Chl-b**

**Units**	**% Fresh Leaves**	**mg/g Fresh Leaves**	**mg/kg Fresh Leaves**	**mg/kg Fresh Leaves**
SEL - reproducibility	0.006	0.028	0.019	0.009
# Samples	133	133	133	133
Outliers	0	0	0	5
Min	0.577	1.534	1.293	0.510
Mean	0.682	2.372	1.632	0.756
Max	0.817	2.968	2.035	0.993
SD	0.056	0.324	0.188	0.124
Segment	4	4	4	4
WL range/step	5000–7144, 7104–10,000/8	5000–7144, 7104–10,000/8	5000–7144, 7104–10,000/8	5000–7144, 7104–10,000/8
Pre-treatments	D2, MSC	D2, MSC	D2, MSC	D2, MSC
Regression method	PLS	PLS	PLS	PLS
Number of factors	7	7	9	10
SEC	0.012	0.132	0.076	0.059
R^2^_cal_	0.983	0.941	0.913	0.899
SECV	0.016	0.155	0.095	0.065
R^2^_cross val._	0.945	0.918	0.883	0.858
NIR repeatability	0.11	0.11	0.13	0.21
DW	1.83	2.00	1.75	1.91
C-Set Durbin-Watson in range 1.5 to 2.5?	yes	yes	yes	yes
Q-value	0.76	0.83	0.79	0.74
RPD_cal_	4.66	2.45	2.47	2.10
RPD_cross val._	3.50	2.09	1.97	1.90

**Table 3.** Statistics of validation during external tests.

**Parameter**	**TKN**	**Total Chl**	**Chl-a**	**Chl-b**

**Units**	**% Fresh Leaves**	**mg/g Fresh Leaves**	**mg/g Fresh Leaves**	**mg/g Fresh Leaves**
# Samples	47	47	47	47
Outliers	0	0	0	0
Min	0.557	1.833	1.275	0.623
Mean	0.678	2.385	1.616	0.760
Max	0.804	2.874	2.058	0.921
SD	0.060	0.262	0.186	0.052
RMSEP	0.028	0.163	0.101	0.104
SEP_-b_	0.028	0.162	0.101	0.101
R^2^_pred_	0.940	0.899	0.845	0.844
RSD	1.415	0.083	0.074	0.020
NIR repeatability	0.11	0.11	0.13	0.21
Bias	0.00	0.018	−0.004	−0.026
Intercept	0.047	0.696	0.384	0.637
Slope	0.924	0.702	0.763	0.167
DW	2.00	1.94	2.39	1.99
V-Set Durbin-Watson in range 1.5 to 2.5?	yes	yes	yes	yes
